# The Impact of the Blood–Brain Barrier and Its Dysfunction in Parkinson’s Disease: Contributions to Pathogenesis and Progression

**DOI:** 10.1021/acsomega.4c06546

**Published:** 2024-11-05

**Authors:** Muhammad Khalid Iqbal, Bakhtawar Khan, Hifsa, Ge YuXuan, Muhammad Mujahid, Mubin Mustafa Kiyani, Hamid Khan, Shahid Bashir

**Affiliations:** † Institute of Brain Disorders, Department of Physiology, Dalian Medical University, Dalian, Liaoning Province 116044, China; ‡ Department of Biochemistry, Government College University, Faisalabad 38000, Pakistan; § Shifa College of Medical Technology, Shifa Tameer-e-Millat University, Islamabad 44000, Pakistan; ∥ Molecular Biology and Bio Interfaces Engineering Lab, Department of Biological Sciences, Faculty of Sciences, International Islamic University Islamabad. H10, Islamabad 44000, Pakistan; ⊥ Neuroscience Center, King Fahad Specialist Hospital Dammam, Dammam 32253, Saudi Arabia

## Abstract

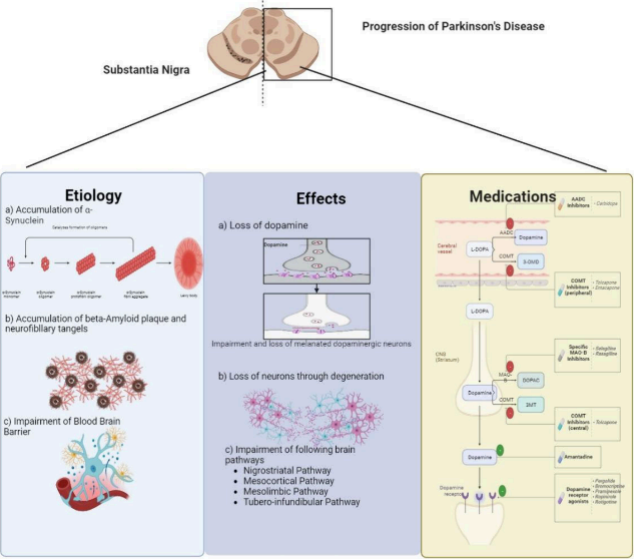

Parkinson’s disease (PD) is a brain disorder in which neuronal cells responsible for the release of dopamine, a neurotransmitter that controls movement, are degenerated or impaired in the substantia nigra and basal ganglia. The disease typically affects people over the age of 5 and presents with a variety of motor and nonmotor dysfunctions, which are unique to each person. The impairment of the blood–brain barrier (BBB) and blood retinal barrier (BRB) due to age-related causes such as weakness of tight junctions or rare genetic factors allows several metabolic intermediates to reach and accumulate inside neurons such as Lewy bodies and α-synuclein, disrupting neuronal homeostasis and leading to genetic and epigenetic changes, e.g., damage to the DNA repair system. This perspective highlights the importance of blood barriers, such as the BBB and BRB, in the progression of PD, as the aggregation of Lewy bodies and α-synuclein disrupts neuronal homeostasis. Genetic and epigenetic factors, neuroinflammation, oxidative stress, and mitochondrial dysfunction play crucial roles in the progression of the disease. The implications of these findings are significant; identifying synaptic dysfunction could lead to earlier diagnosis and treatment, while developing targeted therapies focused on preserving synaptic function may slow or halt disease progression. Understanding the various genetic forms of PD could enable more personalized medicine approaches, and using patient-derived midbrain neurons for research may improve the accuracy of PD models due to the implications of an impaired BBB.

## 1 Introduction

**Figure 1:**
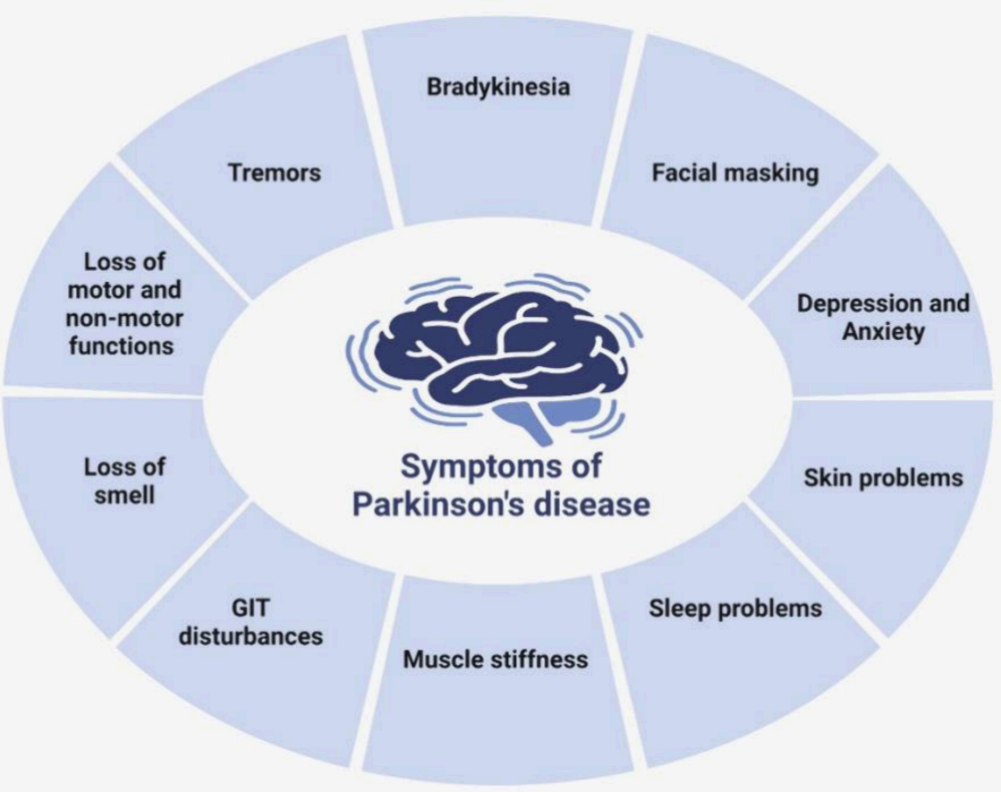
Symptoms of Parkinson’s disease, classifying them into motor and nonmotor symptoms. The loss of motor functions, tremors, muscle stiffness, loss of facial expression (facial masking), and bradykinesia (slow movement) are examples of motor symptoms. Nonmotor symptoms cover a variety of sensations, such as depression and anxiety, anosmia, dermatological issues, gastrointestinal (GIT) disruptions, and sleep difficulties. This detailed depiction showcases the intricate and diverse characteristics of Parkinson’s disease, underscoring the significance of a complete approach to diagnosing and treating the condition.

Parkinson’s disease (PD) is a neurodegenerative disease that affects basal ganglia of the brain, causing impaired and uncontrollable movements. It is the second most common neurodegenerative disease after Alzheimer’s disease. PD is characterized by symptoms such as abnormal gait patterns, bradykinesia, changes in posture, and shortened strides.^1^ Vocal deficits, psychological disturbances, and loss of facial expression are also common signs of PD that have a significant effect on the quality of life. Other symptoms of Parkinson’s disease are depicted in Figure 1.^2^

Parkinson’s disease is unique to each patient and has different symptoms, time of onset, and effects in each case. There are four types of Parkinson’s disease: primary parkinsonism, which occurs in people over the age of 60 due to the degeneration of dopamine producing brain cells and affects body movements only;^3^ dementia associated parkinsonism, where along with impaired motor function, dementia-like symptoms appear; atypical parkinsonism, which occurs in people under the age of 40 and carries its own unique symptoms;^4^ and multiple system atrophy, a rare type of parkinsonism that belongs to atypical parkinsonism and affects both motor as well as nonmotor functions such as heart rate, breathing, memory, and attention.^5^ Other atypical parkinsonism disorders such as dementia with Lewy bodies, progressive supra-nuclear palsy, and corticobasal degeneration are distinct forms of PD that require further research.^6^

The cause of Parkinson’s disease is unknown, but it is believed to be caused by a mix of hereditary factors such as mutations in genes like SNCA, LRRK2, and environmental factors such as exposure to toxins.^7^ PD is diagnosed clinically, as there is no specific test for the disease. Various diagnostic tools, including imaging techniques such as PET, SPECT, TCS, MRI, and thermal imaging, are used to accurately diagnose PD.^8^ In addition, a simple automated framework for PD detection has been proposed, which extracts geometric and texture features from facial visual information, providing a valuable tool for the clinical assessment and screening of PD.^9^ It is important to note that PD is a progressive disease that affects individuals, families, and society, and its prevalence is increasing worldwide.

## 2 Significance of Blood Barriers in Parkinson’s Disease

### 2.1 Role of Blood–Brain Barrier Impairment

The blood–brain barrier is a barricade between the brain and blood circulating in the body with its nutrients, pathogens, and other harmful substances. BBB plays a crucial role in the protection of the brain by maintaining homeostasis and providing protection against toxins and harmful substances by allowing only certain molecules to pass through. It is selectively permeable and allows lipid soluble molecules of size 400–600 Da only to pass through it.^10^

**Figure 2:**
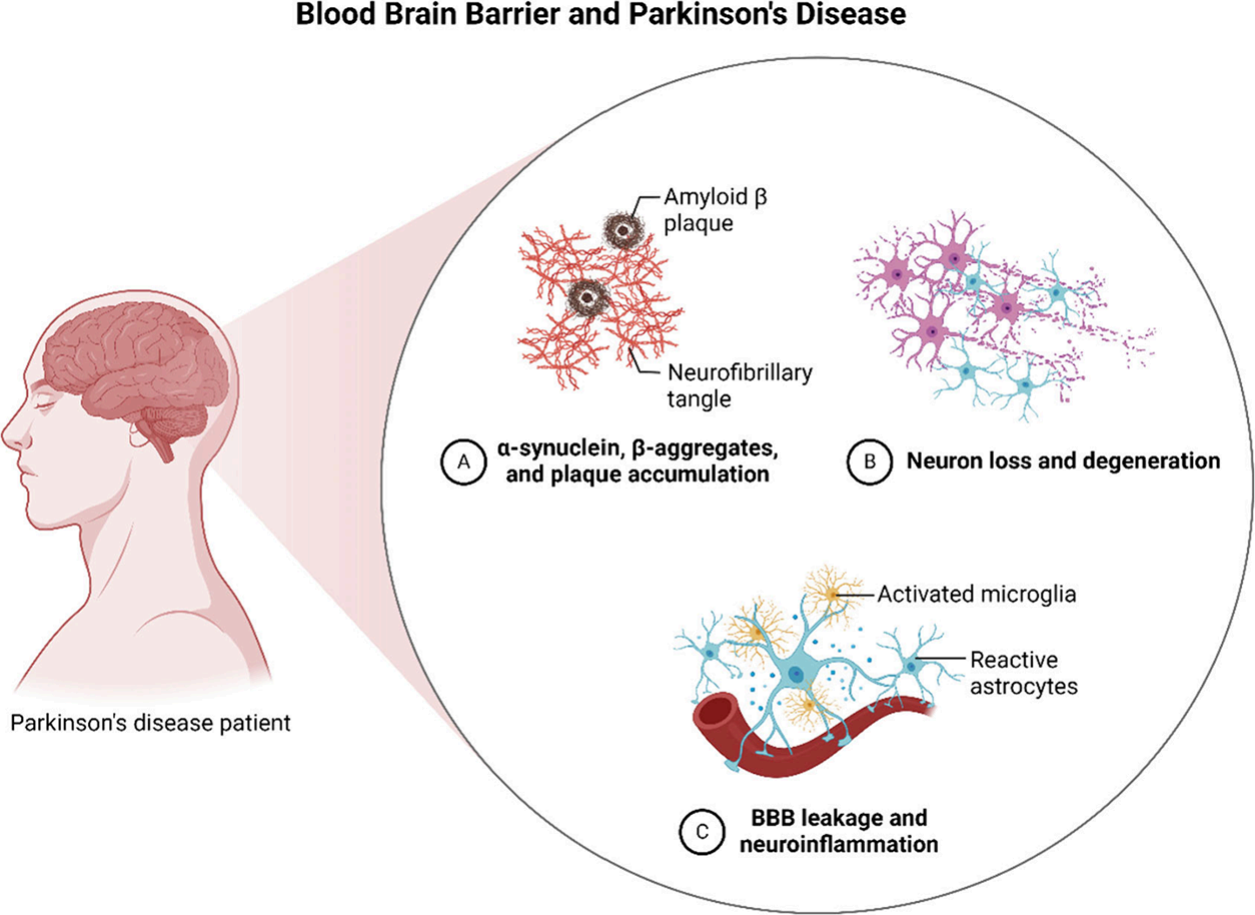
Schematic diagram of the blood–brain barrier (BBB) and its function in Parkinson’s disease. The diagram illustrates the BBB (A) and its impairment in Parkinson’s disease (B), resulting in the buildup of harmful proteins such as amyloid-β plaques, neurofibrillary tangles, α-synuclein, and β-aggregates. Neuroinflammation is worsened by the presence of activated microglia and reactive astrocytes, leading to the loss and degeneration of neurons. The graphic emphasizes the crucial function of the blood–brain barrier (BBB) in obstructing the entry of hazardous compounds into the brain and the severe repercussions that occur when the BBB becomes permeable in Parkinson’s disease.

The blood–brain barrier is a crucial physiological barrier that separates the central nervous system (CNS) from peripheral circulation, protecting its microenvironment. Any type of dysfunction that may occur in the blood–brain barrier is directly associated with various neurological disorders.^11^ Understanding the regulation of BBB function under normal and inflammatory conditions is important. Recent advancements in the in vitro BBB models and cell-specific reporter mice have enhanced our understanding of the BBB dynamics. Strategies to control BBB structure and function have been suggested, and mathematical models have quantitatively correlated BBB anatomical structures with barrier functions. Chemical and physical stimuli can modulate BBB permeability^12,13^ (Figure 2**)**.

### 2.2 Role of Blood–Retina Barrier Impairment

The impaired visual ability in Parkinson’s disease is closely related to the blood–retinal barrier, which regulates the exchange of chemicals between the systemic circulation and retina.^14^ BRB is made up of two barriers, one inner barrier that is similar to BBB and is present in the inner retinal microvasculature and the other outer barrier, which is present at the retinal pigment epithelial cell layer.^15^ The outer barrier is the transportation of nutrients and solutes from blood vessels to the retina protecting it from blood-borne toxins. Both the BBB and the BRB are parts of the neurovascular unit and maintain the normal CNS by regulating the intake and transport of chemicals and solutes as well as steady intercellular interactions.^16^

BRB dysfunction is relevant to PD progression as the sleep disorders and depression cause sleep disturbances and autonomic dysfunction in early and prodromal PD.^17^ The aggregations of α–α-synuclein and degeneration of dopaminergic neuron, leading to motor and nonmotor symptoms, including sleep disorders, are major side effects of PD,^18^ which cause disturbances in BRB. The glymphatic system responsible for optimal sleep and removal of extracellular brain solutes, including α-synuclein, also faces deteriorations due to impaired BBB and enhances α-synuclein accumulation, which leads to dopamine neuron loss as well.^19^ All of these factors contribute to PD pathogenesis and progression.^20^

Disruption of the BRB, observed in various eye diseases, such as diabetic macular edema (DME), can lead to retinal edema and vision loss. Several mechanisms have been identified in the development and maintenance of the inner BRB, including the involvement of tight junction-related proteins and the Wnt signaling pathway.^21,22^ In DME, the breakdown of the BRB is associated with retinal inflammation and the dysregulation of angiogenic factors such as vascular endothelial growth factor (VEGF).^23,24^

### 2.3 Factors Causing Disruptions in the Blood–Brain Barrier

BBB dysfunction plays a significant role in the pathogenesis of Parkinson’s disease.^25,26^ Any damage to BBB allows immune cells and plasma proteins to enter the brain parenchyma, leading to neuro-inflammation.^27^ This neuro-inflammation, triggered by activated glial cells, contributes to neurotoxicity and neuronal dysfunction.^28^ The following section provides a detailed overview of a few factors that cause impairment of the BBB and accelerate the progression of PD.

### 2.4 Accumulation of Protein Aggregates in Brain Microenvironment

The impairment of the blood–brain barrier and reduced expression or weakness of tight junctions cause disruption in BBB integrity in Parkinson’s disease,^29^ which leads to increased vascular permeability and accumulation of Lewy bodies, Lewy body neutrites, and other proteins intermediates, e.g., oligomeric α-synuclein in neuronal cells in the basal ganglia, substantia nigra activated astrocytes.^30^ Neuronal Lewy bodies (LBs) and intrasynaptic aggregation of α-synuclein are hallmarks of brain lesions in PD.^31^ Mutations in genes involved in autophagy and lysosomal pathways, e.g., GBA and ATP13a2, have been linked to lysosomal dysfunction and increased α-synuclein levels.^32^ Abnormalities in ceramide metabolism, which are characteristic of PD, have been found in both brain tissue and the extracellular vesicle (EV) derived from cerebrospinal fluid (CSF). Mitochondrial dysfunction, influenced by mutations in PARK genes, has also been implicated in the formation of Lewy pathology and the aggregation of α-synuclein.^33^

Oligomeric α-synuclein plays a critical role in PD-associated BBB disruption, mediated by astrocyte-derived vascular endothelial growth factor A (VEGFA).^34^ α-Synuclein is responsible for nutrition and the supply of synaptic vesicles to neurons at presynaptic terminals. Their accumulation disrupts cellular homeostasis and causes neuronal death.^35^

High concentrations of homocysteine (Hcy) also induce changes in the homeostasis of the neuronal microenvironment. Homocysteine is an intermediate of methionine metabolism; imbalance in this metabolism can cause irreversible mental illness by damaging the DNA repair system and inducing epigenetic changes, which lead to apoptosis, oxidative stress, excitotoxicity, and other psychiatric disorders, e.g., bipolar disorder and schizophrenia.^36^ These conditions can be ameliorated by ingestion of vitamin B12 and folate, folic acid, and treatment with LCZ696, a novel antihypertensive agent with anti-inflammatory properties.^37^

Similarly, Sphingosine-1-phosphate receptor 2 (S1P2) mediates BBB disruption induced by lipopolysaccharide (LPS) accumulation, which causes systemic inflammation and reduction in tight junction protein expression. The inhibition of S1P2 attenuates neutrophil infiltration and downregulates occludin expression.^38,39^ Additionally, cabergoline, a dopamine D2 receptor agonist, protects BBB integrity against LPS-induced disruption by upregulating zonula occludens-1 (ZO-1).^40^ These findings suggest that BBB disruption is a common pathology in PD and can be influenced by factors such as α-synuclein, Hcy, S1P2, and inflammatory stimuli, highlighting potential therapeutic targets for maintaining BBB integrity in neurodegenerative diseases.

### 2.5 Dis-integrity of Tight Junctions

The breakdown of the blood–brain barrier can occur due to various mechanisms. One mechanism is the damage to the tight junctions, basement membrane, and adhesion molecules, which leads to the disruption of BBB integrity.^41^ Another mechanism is the increased transcytosis and weak tight junctions within autonomic nuclei, allowing the entrance of plasma constituents into the brain parenchyma.^42^

Furthermore, the age-related decline in BBB integrity may be associated with the manipulation of integrin function, as blocking β1 integrin has been shown to amplify hypoxia-induced vascular disruption and BBB breakdown.^43^ Understanding these mechanisms contributing to BBB breakdown is crucial for developing strategies to maintain the BBB integrity and prevent the entry of harmful substances into the brain.

## 3 Neuro-inflammation

Neuro-inflammation plays a significant role in the breakdown of the blood–brain barrier and the pathophysiology of Parkinson’s disease.^27,44^ It is caused by viral infection, stress, living conditions, autoimmune disorders or underlying inflammatory processes, e.g., activation of glial cells, such as microglia and astrocytes, and triggers the release of pro-inflammatory cytokines, leading to neurotoxicity and neuronal dysfunction.^45^ Inflammatory mediators released by perivascular cells, such as microglia and astrocytes, can disrupt the BBB and amplify neuro-inflammation.^46^ Additionally, infiltrating blood-borne immune cells, including neutrophils, monocytes, and T lymphocytes, increase BBB permeability and contribute to microvascular disorder and inflammation.^47^ The infiltration of these immune cells is not solely a consequence of BBB failure but is facilitated by various mediators produced by the neurovascular unit.^48^

Impairment of the BBB caused by any type of factor allows immune cells and plasma proteins to enter the brain parenchyma, amplifying neuro-inflammation.^49^ Increased permeability of the BBB to neurotoxic substances leads to PD pathogenesis,^25,27,50^ which causes neuro-inflammation, neurotoxicity, and neuronal dysfunction, which lead to the entry of immune cells or plasma proteins into the brain parenchyma.^51^ Additionally, chronic gut inflammation can lead to a leaky gut and systemic inflammation, which can further contribute to neuro-inflammation and neurodegeneration via BBB permeability.^52^ In PD, BBB disruption allows the trafficking of neurotoxic substances into the brain, contributing to the degeneration of dopaminergic neurons and the progression of the disease.^53^

In PD, abnormal aggregation of α-synuclein activates toll-like receptor 4 (TLR4), releasing pro-inflammatory cytokines and causing fatigue symptoms.^54^ Chronic peripheral inflammation and immune activation responses induce elevated levels of pro-inflammatory cytokines, which can cross the BBB and contribute to the occurrence of fatigue. Understanding the inflammatory mechanisms involved in BBB breakdown and neuro-inflammation can provide insights for the development of targeted treatments for PD. The knowledge of these underlying mechanisms of BBB impairment by inflammatory mediators released by perivascular cells is crucial for developing novel treatments for neuro-inflammatory diseases; e.g., modulating the gut microbiota through interventions like probiotics and fecal microbiota transplantation (FMT) may help restore gut dysbiosis, reduce inflammation, and potentially modulate the clinical phenotype of PD.

### 3.1 Oxidative Stress and Mitochondrial Dysfunction

Oxidative stress and mitochondrial dysfunction are seen in Parkinson’s disease and contribute to blood–brain barrier breakdown.^55,56^ Mitochondrial dysfunction, characterized by decreased ATP levels, disrupted mitochondrial morphology, and altered mitochondrial function, has been observed in neuronal cells exposed to particulate matter (PM) and lead (Pb).^57,58^ Additionally, PM exposure has been shown to increase oxidative stress and inflammatory cellular damage, leading to mitochondrial disruption and neurotoxic effects in neuronal cells.^59^

Mitochondrial alterations have also been observed in Rett syndrome (RTT), a neurodevelopmental disorder, and the absence of the MECP2 gene in RTT may lead to altered mitochondrial function and elevated levels of cellular oxidative stress.^60^ Additionally, mutations in the glucocerebrosidase (GBA) gene, which is associated with PD, can also contribute to mitochondrial dysfunction and altered lipid homeostasis. Furthermore, PGC-1α downregulation has been observed in animal and cellular models of neurodegenerative diseases, suggesting its role in the pathophysiology of PD.^35^ Therefore, the transcriptional coactivator peroxisome proliferator-activated receptor α-coactivator 1-alpha (PGC-1α) has been implicated in maintaining mitochondrial quality control and neuronal survival.

### 3.2 Other Associated Disorders That Enhance Progression of Parkinson’s Disease

The breakdown of the blood–brain barrier and dysfunction of tight junction proteins are important mechanisms under various neurological conditions. Implications of the blood–brain barrier (BBB) dysfunction in Parkinson’s disease (PD) pathogenesis include alterations in nutrient transport and waste clearance. The gut microbiota has been implicated in the pathogenesis of PD, and gut microbial dysbiosis may contribute to the loss of dopaminergic neurons through mitochondrial dysfunction.^61^ Similarly, endothelial dysfunction is considered an etiological factor in inflammatory bowel disease (IBD), and it can lead to structural and functional changes in the vascular endothelium, including alterations in nutrient transport and waste clearance.^62^ Inflammatory bowel disease (IBD) can cause loss-of-function mutations in the PTPN2 gene, which lead to increased intestinal permeability, that may also be relevant to BBB dysfunction in PD.^27^ Similarly, chronic inflammation in conditions like sleep apnea and Alzheimer’s disease can affect the integrity of the BBB, resulting in increased permeability and decreased expression of tight junction proteins.^63^

**Figure 3:**
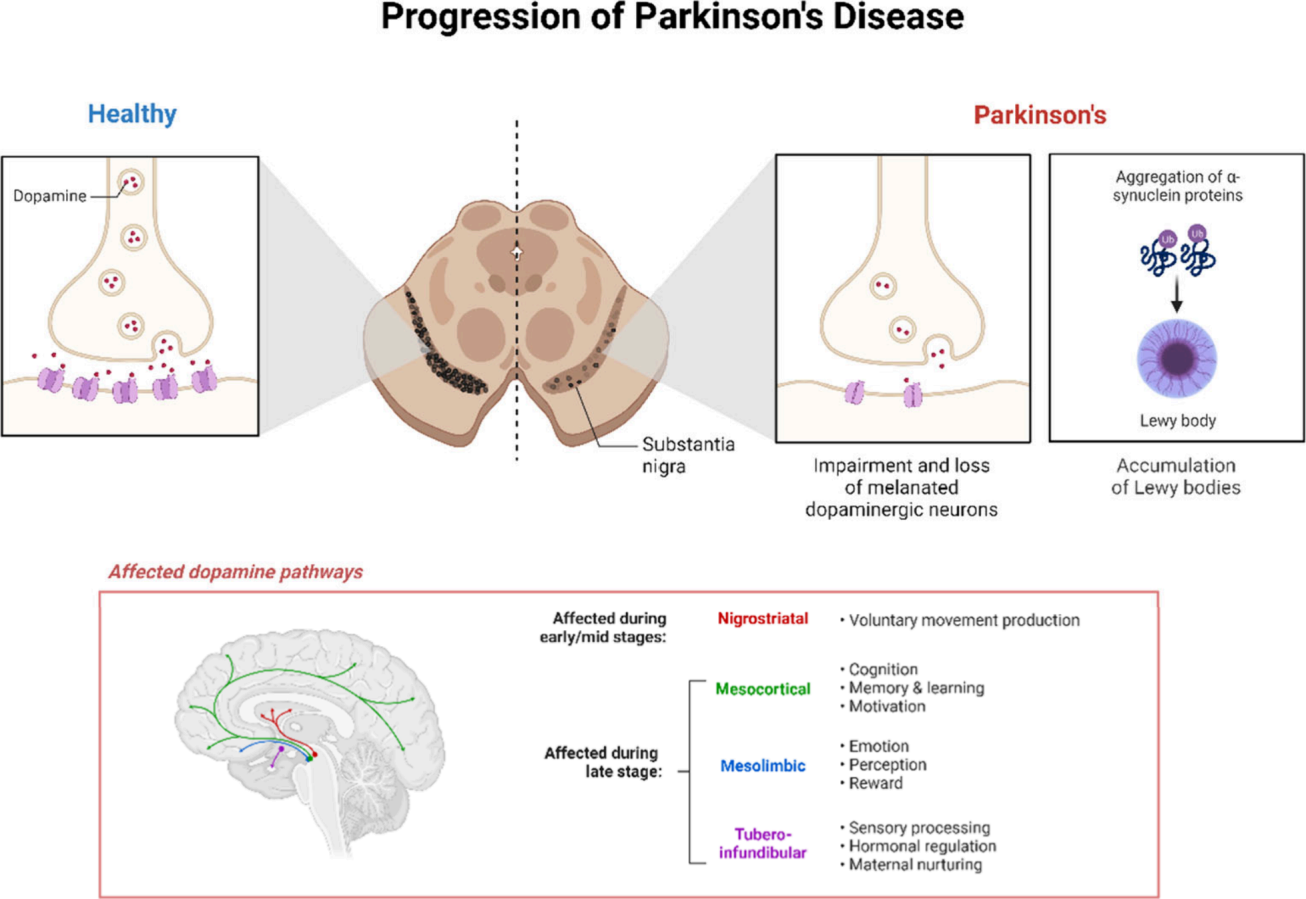
Sequential development of neuropathological changes in Parkinson’s disease, starting from a healthy brain and leading to the appearance of the disease. The accumulation of dopamine and the creation of Lewy bodies cause deterioration of dopaminergic neurons in the substantia nigra. This disrupts both motor and nonmotor pathways, such as the nigrostriatal, mesocortical, mesolimbic, and tubero-infundibular pathways, ultimately leading to the appearance of symptoms.

One study found that severe hypoglycemia leads to cognitive dysfunction in diabetic mice, and this is related to pericyte dysfunction and BBB destruction.^64^ Other health conditions, e.g., disruptions in circadian rhythm, ischemic stroke, and aneurysmal subarachnoid hemorrhage, lead to impaired BBB, weak tight junctions, inflammation, and oxidative stress in the brain^65−67^ (Figure 3).

## 4 Diagnosis of Parkinson’s Disease

The diagnostic techniques include noninvasive imaging techniques, e.g., magnetic resonance imaging techniques, such as dynamic contrast-enhanced and dynamic susceptibility contrast MRI, and can be used to assess BBB integrity by detecting leakage of contrast agents. Other MRI techniques target different aspects of the BBB and use endogenous markers like water and glucose as contrast media.^68^ These techniques provide insights into the structural and biochemical changes associated with PD and can help identify biomarkers of disease progression. Other imaging techniques like position emission tomography (PET), single-photon emission computed tomography (SPECT), transcranial sonography (TCS), and thermal imaging can also be used to diagnose PD and assess autonomic dysfunction.^69^ These imaging techniques offer a more accurate and sensitive diagnostic tool for PD, improving the management and treatment of the disease. Further research and development of these noninvasive imaging techniques hold promise for better understanding and treatment of PD.

Olfactory dysfunction, such as hyposmia, can be a sensitive marker for early diagnosis of PD and may provide insights into the underlying mechanism of Lewy neurodegenerative diseases.^70^ Similarly, deficits in short-term visual memory in patients with REM sleep behavior disorder (RBD) may serve as a marker for early PD and could be used in clinical trials for novel disease interventions.^71^

## 5 Medications

The medications for PD available at the moment cannot completely cure the disease and are used to provide relief from symptoms associated with the disease. There are two types of medications available for PD: dopaminergic drugs that work through dopamine pathways and nondopaminergic drugs, e.g., cholinergic inhibitors that work on other pathways. Both of these types function in separate ways and enhance dopamine levels in the neurons as described in Table 1.

**Table 1:** Medications of Parkinson’s Disease, Their Mode of Action, and Side Effects

medications	mode of action	side effects	refs
Levodopa	crosses BBB and converts into dopamine by DOPA decarboxylase enzyme	anxiety, hallucinations, dyskinesia, and gastrointestinal disturbances	(72)
decarboxylase inhibitors	administered in combination with levodopa and blocks the conversion of levodopa outside the CNS into dopamine to reduce unwanted effects, e.g., bradykinesia	hallucinations, dizziness, trouble sleeping, and nausea	(73)
(a) Carbidopa			
dopamine antagonists	activate dopaminergic pathways by binding to dopamine receptors	nausea, dry mouth, hallucinations, sleepiness, constipation, edema, and development of impulsive control disorder	(74), (75)
(a) Ropinirole			
(b) Pramipexole			
(c) Rotigotine			
monoamine oxidase (MAO) inhibitors	MAO inhibitors reduce the breakdown of dopamine and enhance availability of the neurotransmitter	joint pain, fatigue, insomnia, dizziness, nightmares, hallucinations, headache, and indigestion	(76), (77)
(a) Selegiline			
(b) Azilect			
catechol-*O*-methyl transferase (COMT) inhibitors	COMT inhibitors reduce dopamine breakdown and help in controlling motor movements	amplification in dyskinesias, hepatotoxicity, sleepiness, gastrointestinal disturbances, hallucinations, chest pain, and urine discoloration	(78), (79)
(a) Opicapone			
(b) Entacapone			
(c) Tolacapone			
amantadine	the mode of action of amantadine is not clear; however, it is believed that it may act as a weak glutamate antagonist; it reduces muscle stiffness, tremors, and fatigue and levodopa-induced dyskinesia	sweating, agitation, headache, gastrointestinal disturbances, swelling in legs, hallucinations, blurred vision, loss of concentration, nightmares, and loss of appetite	(80), (81)
(a) Symmetrel			
cholinergic inhibitors	reduce activity of acetylcholine in basal ganglia and increases dopamine uptake and storage in neurons; it improves rigidity and tremors and restores balance between dopamine and acetylcholine	cognitive impairment, confusion in elderly, hallucinations, blurred vision, and gastrointestinal disturbances	(82), (83)
(a) Procyclidine			
(b) Orphenadrine			
(c) Benztropine			
(d) Trihexyphenidyl			

### 5.1 Emerging Therapeutic Strategies for Parkinson’s Disease

Current therapies for PD include dopaminergic therapy and symptom management. However, these therapies have limitations and fail to address nonmotor and nondopaminergic aspects of the disease. There is a critical need for dopaminergic therapies with minimal side effects, treatment for nonmotor symptoms, and disease-modifying therapies. While existing treatments focus on restoring dopaminergic function and managing symptoms, they do not target the underlying neurodegenerative processes. Novel therapies are being explored to address cell death and disease progression. Astrocytes, which play a role in maintaining neuronal environment and exert neuroprotective effects, have been identified as a potential target for neuroprotection in PD.^84^ Experimental approaches targeting astrocytes have shown promise in preventing dopaminergic neurodegeneration. However, more research is needed to fully understand the implications and potential of targeting BBB integrity as a therapeutic strategy in PD.

Other potential therapies include restoring barrier function in Parkinson’s disease; e.g., defects in epithelial membrane barriers in the gut and cerebral vasculature can increase vulnerability to external factors involved in PD pathogenesis.^85^ Enteric glial cells (EGCs) play a major role in PD-related gastrointestinal disturbances and central disease development.^86^ Impairment of barrier permeability triggers dysfunctions of EGCs and reactive gliosis, leading to neuro-inflammation and pathological changes in the enteric nervous system.

Novel therapy approaches for PD aim to target symptoms, halt pathology, minimize neuronal loss, and moderate disease progression. The pathogenesis of PD involves α-synuclein aggregation, oxidative stress, ferroptosis, mitochondrial dysfunction, neuro-inflammation, and gut dysbiosis.^87^ Future interventions may include therapies that restore barrier function, avoid disruption of the intestinal epithelial barrier, and prevent reactive gliosis and neuro-inflammation. Additionally, noninvasive music therapy techniques have shown potential in improving motor, speech, and cognitive skills in PD patients.

The in vitro BBB models are being used widely to study the barrier function of the BBB. These models simulate the working principle of BBB and can be used to quantify its permeability to water, ions, and solutes.^11^ Assessing the permeability of small molecules through the barrier is important for the development of central nervous system drugs. Other in silico and in vitro approaches, such as the parallel artificial membrane permeability assay (PAMPA-BBB) and computational methods, are also under investigations to predict BBB permeability in the early stages of drug discovery.^88^

Novel strategies, such as the use of cell-penetrating peptides (CPPs), are being explored to facilitate drug delivery across the BBB. CPPs have the potential to serve as shuttles for brain-specific drugs. Photobiomodulation (PBM) with near-infrared (NIR) light has shown potential as a noninvasive therapeutic approach for neurological disorders. NIR light irradiation has been found to increase the permeability of in vitro BBB models by affecting mitochondrial activity, reactive oxygen species (ROS) levels, and the expression of tight junction proteins.^89^ Nanoparticle-based drug delivery systems, such as solid lipid nanoparticles (SLNs) and nanostructured lipid carriers (NLCs), have been investigated for their potential in facilitating drug transport across the BBB. Carriers have advantages of delivering hydrophilic and hydrophobic agents to the brain for the treatment of neurodegenerative diseases and brain cancers.^90^

Advances in drug delivery across the blood–brain barrier have significant therapeutic implications and future potential in Parkinson’s disease treatment. Nano drug delivery systems, such as solid lipid nanoparticles (SLNs), PLGA nanoparticles (NPs), and graphene oxide (GO) nanosheets, have shown promise in delivering therapeutic agents to the brain.^90^ These systems offer controlled drug delivery, a longer circulation time, target specificity, and reduced toxicity. Additionally, receptor-mediated transcytosis (RMT) has been utilized to transport nanoparticles across the BBB.^91^ The development of disease-modifying therapies and treatments for motor complications in PD is also progressing. Nanoparticles have the potential to improve the pharmacokinetics of conventional therapies and deliver better therapeutic agents.

## 6 Future Research Directions and Challenges

The therapeutic implications and future potential of research directions and challenges related to Parkinson’s disease are being explored. Medical imaging, such as magnetic resonance imaging (MRI), is being used to develop support systems for the diagnosis and prognosis of PD. The functional organization of thalamic inputs to the basal ganglia, including the intralaminar nuclei, is of particular interest in understanding motor and nonmotor functions in PD.^92^ Angiotensin-II AT1 receptor blockers (ARBs) show potential beneficial effects in PD patients, and further clinical trials are warranted.^93^ Emerging evidence suggests that disrupted oscillatory activity in cortico-basal ganglia-thalamo-cortical (CBGTC) and cerebellar networks can be partially corrected by applying deep brain stimulation (DBS).^94^ Similarly, intracerebro-ventricular administration of platelet-derived growth factor-BB has shown promising results in restoring function in Parkinson’s disease, including restoration of striatal dopamine transporter binding sites and expression of nigral tyrosine hydroxylase.^95^ The study of the placebo effect in PD has identified genuine psychologic placebo effects and nocebo responses, which have important implications for clinical trial design and drug dosage. Recent clinical trials have focused on novel strategies targeting α-synuclein and repurposing drugs for disease modification in PD, but results have been disappointing.^96^ Future research directions should continue to explore these therapeutic targets and address the challenges of the BBB in PD.

## 7 Conclusion

Parkinson’s disease is a slow and progressive neurodegenerative disorder. It begins around 10 years before the manifestation of symptoms and affects dopaminergic pathways only. It gradually creates an imbalance between two important neurotransmitters, dopamine and acetylcholine, leading to motor and nonmotor dysfunction. PD occurs due to genetic and environmental factors and depends on a person’s living conditions. It is directly connected to the blood–brain barrier as alterations to the BBB can accelerate PD progression. The weakening of the BBB gives rise to the accumulation of Lewy bodies, Lewy neurites, and protein aggregates, inducing neuro-inflammation that affects the BRB and causes impairment of vision, muscle rigidity, slow and involuntary movements, changes in speech, and other unique symptoms in each patient. Medications available for PD are used to relieve symptoms and slow disease progression. Therefore, it is essential to understand the mechanisms underlying PD and the effect of BBB impairment.

Regarding treatments for PD, significant developments have been made in the fields of regeneration, gene therapy, and stem cell approaches. However, there is a need for more drug repurposing, as they are safe to use and previously available medicines have limited effects. These medications can only slow progression, require constant dose increase, and lead to tolerance development in patients. Eventually, increased dose can result in side effects such as blurry vision, dementia, confusion, hallucinations, loss of cognitive function, loss of appetite, and other gastrointestinal disturbances.

## Data Availability

This is a review article, and all references supporting the findings discussed are available in the reference list.
